# Acetylcholine Use in Modern Cardiac Catheterization Laboratories: A Systematic Review

**DOI:** 10.3390/jcm11041129

**Published:** 2022-02-21

**Authors:** Andrea Marrone, Rita Pavasini, Ennio Scollo, Federico Gibiino, Graziella Pompei, Serena Caglioni, Simone Biscaglia, Gianluca Campo, Matteo Tebaldi

**Affiliations:** Cardiology Unit, Azienda Ospedaliero-Universitaria di Ferrara, 44124 Cona, FE, Italy; mrrndr1@unife.it (A.M.); pvsrti@unife.it (R.P.); sclnne@unife.it (E.S.); gbnfrc@unife.it (F.G.); pmpgzl@unife.it (G.P.); cglsrn@unife.it (S.C.); bscsmn@unife.it (S.B.); cmpglc@unife.it (G.C.)

**Keywords:** acetylcholine provocation test, coronary spasm, diffuse spasm, prognosis, vasospastic angina, safety

## Abstract

Background: The use of acetylcholine for the diagnosis of vasospastic angina is recommended by international guidelines. However, its intracoronary use is still off-label due to the absence of safety studies. We aimed to perform a systematic review of the literature to identify adverse events related to the intracoronary administration of acetylcholine for vasoreactivity testing to fill this gap. Methods and results: We conducted a systematic review of observational studies and randomized controlled trials dealing with the intracoronary administration of acetylcholine. Articles were searched in MEDLINE (PubMed) using the MeSH strategy. Three independent reviewers determined whether the studies met the inclusion and exclusion criteria. A total of 434 articles were selected. Data concerning clinical characteristics, study population, acetylcholine dosage, and adverse effects were retrieved from the articles. Overall, 71,566 patients were included, of which only 382 (0.5%) developed one adverse event, and there were no fatal events reported (0%). Conclusions: Intracoronary administration of acetylcholine in the setting of coronary spasm provocation testing is safe and plays a central role in the evaluation of coronary vasomotion disorders, making it worthy of becoming a part of clinical practice in all cardiac catheterization laboratories.

## 1. Introduction

More than 50 years ago, Prinzmetal first described “variant angina” as a kind of chest pain occurring at rest, with electrocardiographic (ECG) evidence of ST segment elevation due to increased coronary tonus. This condition, now called vasospastic angina (VSA), is caused by a vasomotor disorder in which coronary vascular hyper-reactivity and vasoconstrictor stimuli lead to vascular spasm that generates ischemia [1]. VSA may be associated with coronary atherosclerosis (as it promotes vaso-reactivity) or microvascular disease, although it can also occur alone as the sole cause of angina [2]. VSA is associated with major adverse events, such as sudden cardiac death [3], acute myocardial infarction (MI) [4], and syncope [5]. Despite the fact that therapies with a prognostic impact that are effective in reducing vasoconstriction are available, the methods available for diagnosis are very limited [6].

Coronary angiography provides information mostly on the morphology of epicardial vessels; however, it has very low sensitivity for the identification of VSA, the latter requiring further diagnostic assessment, such as spasm provocation testing.

Intracoronary infusion of vasoactive agents (acetylcholine, ergonovine, and substance P) allows for the assessment of coronary vascular function. This represents the gold standard for the diagnosis of VSA, with more than 90% sensitivity and 99% specificity. In particular, intracoronary (IC) administration of acetylcholine (ACh) is the most common method for assessing vascular reactivity. Patients presenting with signs and symptoms of angina or myocardial infarction in the absence of significant stenosis in at least one epicardial coronary artery remain at risk of an adverse cardiovascular outcome without adequate diagnosis and treatment. It has been amply demonstrated that angiographically insignificant stenoses can be significant in functional assessment and vice versa. For this reason, it is crucial for modern cath labs to introduce routine hyperemic (FFR) and non-hyperemic (iFR, RFR, etc.) functional assessments. Once epicardial disease has been excluded, it is important to perform a thorough evaluation of the coronary microcirculation using both the coronary flow reserve (CFR) and the index of microcirculatory resistance (IMR) before moving on to the evaluation of vascular reactivity.

Acetylcholine is a parasympathetic nervous system transmitter that binds to both nicotinic and muscarinic receptors, playing a central role in vascular tone regulation. In the case of a healthy vascular system, a predominant role of the endothelium over smooth muscle cells is responsible for endothelial-dependent vasodilation, whereas, in the case of vascular dysfunction, the cell-mediated vasoconstriction of smooth muscle overcomes vasodilation [7]. The use of ACh for provocation testing has been used for a long time, and more recently, several evidence-based indications for the diagnosis of VSA have been formulated by the Coronary Vasomotion Disorders International Study (COVADIS) Group [1] and by the Japanese Circulation Society (JSC) group with the Guidelines for Diagnosis and Treatment of Patients with Vasospastic Angina (Coronary Spastic Angina) [8]. However, in some countries, such as Italy, safety concerns still persist about intracoronary administration of ACh, limiting its widespread use in clinical practice. As a matter of fact, intracoronary administration of ACh is still off-label in western countries, limiting its diagnostic and prognostic potential. All of this means that the operator has to use the drug through a route of administration different than the one described in its instruction, which does not encourage its use throughout modern cath labs.

The aim of our systematic review was to identify and appraise previous studies in which spasm provocation tests with the intracoronary infusion of ACh are reported, and to describe the types and the incidence of side effects eventually related to ACh administration in a large population.

## 2. Methods

We developed a systematic review following the preferred reporting items for systematic reviews and meta-analysis guidelines (PRISMA) [9,10,11,12]. The protocol registration application for this study was performed on an international prospective register for systematic reviews (PROSPERO) on 8 November 2021 with ID number CRD42021289721. Using the MeSH strategy in MEDLINE (PubMed), three operators (A.M., E.S., and F.G.) independently and systematically searched studies about patients with acute or chronic coronary syndrome with and without evidence of obstructive epicardial coronary artery disease and needing functional assessment with the use of intracoronary ACh during coronary angiography to test the presence of coronary artery spasm. The terms searched were (acetylcholine OR (acetylcholine testing)) AND ((angina) OR (vasospasm) OR (vasospastic angina)). The research was conducted in November 2021.

The inclusion criteria were (i) studies including the use of intracoronary ACh for testing coronary epicardial spasm. The exclusion criteria were (i) duplicates; (ii) reviews or editorials; (iii) meta-analyses; (iv) duplication of the sample population; (v) studies on animals; (vi) grey literature; (vii) abstracts or posters. Only papers published in English and in peer-reviewed journals were retrieved.

The three operators completed a database with the data regarding the journal, study type, year of publication, indication for ACh administration, diagnosis, dosage of ACh, route of administration, and side effects related to ACh administration. The main purpose of the systematic review was to describe the use of ACh in cath labs in terms of dosage administration, route of administration, and side effects. The quality of the included studies was tested using pre-specified electronic forms of MINORS criteria [13], with overall scores ranging between 10 and 18 (Table S1).

Patient data and clinical characteristics, as well as procedural features, were expressed in numerical form, categorical variables were expressed as a percentage, and continuous variables as means. Comparisons between categorical variables were carried out with Pearson Chi-square. One- or two-tailed tests were employed as appropriate, and the statistical significance was defined as *p* < 0.05. All analyses were performed with STATA (version 16.0).

## 3. Results

### 3.1. Search Strategy

**Figure 1:**
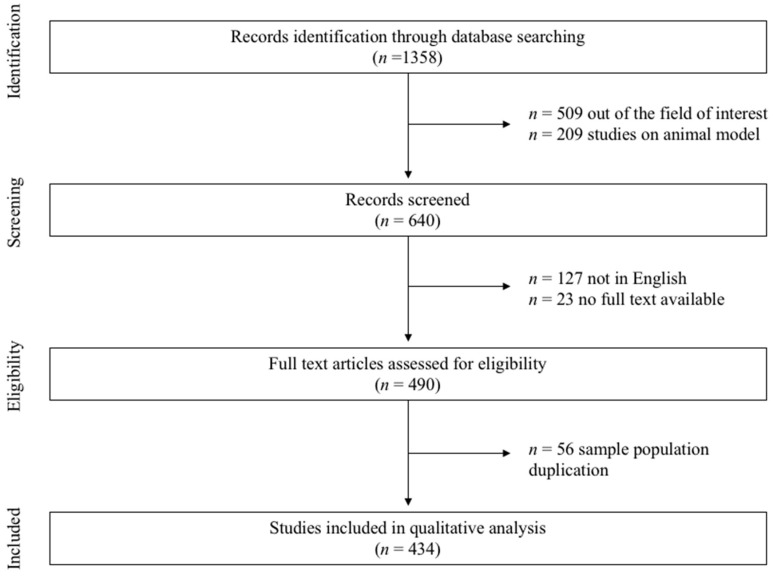
Outline of the search strategy.

Overall, 1358 records were identified through database searching. After analyzing the titles, abstracts, and the application of the inclusion and exclusion criteria, 924 records were excluded because they were not focused on the item of interest. Finally, 434 articles were included in this systematic review; 15 of them were randomized clinical trials, while 419 were observational studies (Figure 1). A detailed list of the RCTs included is provided in Table 1.

### 3.2. Adverse Events Related to ACh Administration

**Figure 2:**
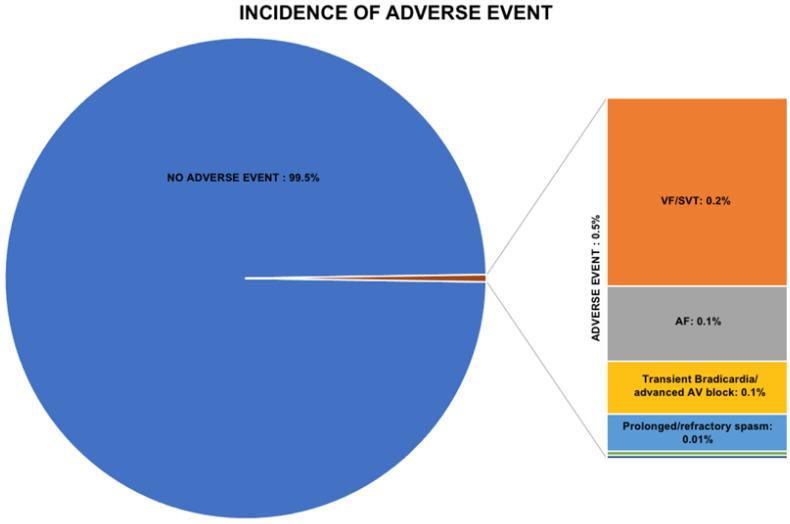
Incidence of adverse events. AF: atrial fibrillation; AV: atrial ventricular, MI: myocardial infarction, NSVT: non-sustained ventricular fibrillation, SVT: sustained ventricular fibrillation; VF: ventricular fibrillation.

Adverse events were categorized as major or minor. Major adverse events included ventricular fibrillation (VF) and sustained ventricular tachycardia (SVT), cardiogenic shock, myocardial infarction (MI), and prolonged/refractory spasm. Minor adverse events included transient bradycardia and advanced atrio-ventricular (AV) block, atrial fibrillation (AF), non-sustained ventricular tachycardia (NSVT), and hypotension. Overall, 382 (0.5%) of 71,566 patients developed at least one adverse event. Among 1521 patients in the randomized controlled trials, 17 (1.1%) experienced adverse events, while among 70,045 patients of observational studies, adverse events were reported in 365 (0.5%). Of note, there were no reported deaths in the overall population (Table 2 and Figure 2). The most common adverse event was VF/SVT, followed by AF. The doses of intracoronary ACh ranged between 20 and 100 mcg for LCA, with a maximum dose of 200 mcg, and between 20 and 50 mcg for RCA, with a maximum dose of 80 mcg (a complete list of definitions is provided in the Supplementary Materials).

### 3.3. INOCA vs. MINOCA

Of the 434 selected articles, 145 involved INOCA (ischemia and non-obstructive coronary artery disease) patients, 112 involved MINOCA (myocardial infarction and non-obstructive coronary artery disease) patients, while the remaining 177 studies included a mixed population. Considering only INOCA patients, out of 38,987, only 195 (0.5%) experienced an adverse event related to ACh administration, while the incidence rate in the MINOCA population (34,319 pts) was 0.1%, configuring a highly significant difference among the two groups (*p* < 0.00001).

## 4. Discussion

The main findings of the present systematic review are as follows:

1. The intracoronary use of ACh has been tested in a wide number of studies (specifically, 434).
2. The total number of reported side effects is low, occurring in 0.5% of the patients included.
3. The most frequent side effects are arrhythmic events (VF/SVT and AF occurring in 0.2% and 0.15% of the overall population, respectively).
4. Ach-related death has never been described.
5. Patients with MINOCA have a significantly lower number of events than INOCA patients when assessed for ACh.

MINOCA comprises 5–20% of all type 1 AMI cases. There are several pathophysiological mechanisms for MINOCA. The causes of MINOCA can be classified as epicardial or microvascular. Regarding the former, about 40% of patients with MINOCA have coronary plaque disease, such as plaque rupture and erosion; 25% of the patients, especially women, present with coronary artery dissection. The prevalence of coronary artery spasm as a cause of MINOCA is variable, ranging from 5 to 95% of these patients. As for microvascular etiology, coronary microvascular spasm, Takotsubo cardiomyopathy, myocarditis, and coronary thromboembolism are the most common causes [28,29].

The present evaluation shows a low rate of side effects, in line with the individual experiences reported in the literature. Ciliberti et al. [30] showed that the frequency of major complications ranged from 0% to 4.9%, while the rate of minor complications ranged from 0% to 16.3%. Among the major complications described in the existing literature, the most common is ventricular fibrillation (VF) or sustained ventricular tachycardia (SVT) occurring in 0.69% of the cases, while shock (0.03%), myocardial infarction (0.01%), and prolonged/refractory spasm (0.01%) are quite rare. As both the sinus and atrioventricular node blood supply is provided by the RCA (or circumflex in the case of left-dominant coronary circulation), transient bradycardia or advanced atrioventricular block are two of the most common minor complications (2.71% of the cases), together with paroxysmal atrial fibrillation (1.55%), non-sustained VT (0.91%), and hypotension (0.03%) [30]. Despite guidelines outlining the benefit of a temporary pacemaker during the procedure [31], the placement of the pacemaker itself has some risks; hence, many authors, such as Ford et al. stated that the use of this device is not routinely needed unless RCA is infused [7]. Isogai et al. assessed that the rate of serious complications was less than 1% in a large cohort of 21,512 patients undergoing pharmacological provocation testing during coronary angiography [32]. Their results were concordant with those of other international authors reporting a cardiac complication rate of 0.5% [33,34,35,36]. In a multicenter registry study of the Japanese Coronary Spasm Association, a total of 1244 patients underwent a provocative test with acetylcholine or ergonovine, with a rate of arrhythmias around 3.2% with acetylcholine. Furthermore, this study showed a strong correlation between diffuse RCA spasm and the occurrence of ventricular tachycardia or ventricular fibrillation [32].

Safety data regarding high-dose acetylcholine were provided by Sueda et al., who investigated the clinical usefulness and safety of the maximal ACh dose of 200 mcg into the LCA in Japanese patients with and without ischemic heart disease, compared with those who received a lower dose of 100 mcg. Provoked positive spasm as well as chest symptoms, ECG changes, LAD, and diffuse spasm were higher with the 200 mcg of ACh. A higher dosage of intracoronary ACh was found not to be responsible for adverse events, as the rate of complications was similar to those at lower doses. Hence, not only is a higher dose of intracoronary ACh useful, but it was also confirmed to be safe in the induction of coronary artery spasm [37].

Further proof of this is that over the years, several international guidelines took up the question of correct indications for provocative spasm testing (Table 3).

According to the JCS Guidelines, patients undergoing invasive provocative testing should withdraw calcium channel blockers and long-acting nitrates 48 h prior to the examination. After baseline coronary angiography, provocation coronary testing is performed with IC administration of 20, 50, or 100 mcg in a 37 °C physiological saline solution into the LCA over a period of twenty seconds, while a reduced dose of 20 or 50 mcg is administered into the RCA with a 3 to 5-min interval between injections. In case no coronary spasm is provoked, an increased dose of 80 mcg in the RCA and 200 mcg in the LCA can be administered. In case of persistent coronary spasm or hemodynamic instability, IC administration of 2.5–5.0 mg isosorbide dinitrate is recommended. It is also recommended that the ECG and blood pressure are monitored during the entire procedure, with a 12-lead ECG performed every 30 s [31]. The growing interest in the intracoronary administration of ACh and other vasoactive agents has somehow caused European and US authors to develop diagnostic protocols and consensus documents over the years. Ford et al., in their review, provided a procedural approach in which the first step is endothelial function assessment (“ACh challenge”) with ACh infusion at a concentration of 10−6 mol/L at 1 mL/min for 2 min. In the case of a positive test (usually chest pain, ST segment deviation, >90% vasoconstriction), coronary endothelial-independent function with intracoronary administration of 300 mcg (3ml) of glyceryl trinitrate (GTN) bolus has to be evaluated. In the case of a negative test, an incremental ACh challenge can be performed with the infusion of 10^−5^ mol/L (1.82 mcg/mL) of ACh at 1 mL/min for 2 min with careful assessment of the patient’s ECG and blood pressure. After repeating the angiography, in the case of negative results, incremental doses of ACh up to a concentration of 10^−4^ mol/L (18.2 mcg/mL) are recommended. In patients showing a physiological response to ACh infusion at 10^−4^ mol/L, a final spasm provocation test with 100 mcg of ACh over 20 s has to be performed, repeating the angiography afterward. Finally, the coronary endothelial independent function assessment is performed as described above [38]. Data regarding dosing regimens are variable in the literature. Hence, the development of a precise dosing regimen remains controversial.

Several studies investigated gender differences in the variability of the IC ACh response. Men showed a higher dose-independent response to acetylcholine administration and a dose-response relationship between ACh and the minimal lumen diameter (MLD) with doses greater than 200 mcg, while women showed minimal variation in the MLD with doses higher than 50 mcg. This difference may be due to tortuous coronary arteries with smaller diameters and thinner walls in women and/or sex-related differences in muscarinic receptors [40,41].

These data show that ACh-based provocative tests not only are safe, but they also play a central role in the assessment of coronary vascular function, which is critical nowadays for the diagnosis of VSA, a condition associated with a poor quality of life and prognosis and for which therapies are available that have an impact on mortality and morbidity [42,43]. Despite this, widespread skepticism still persists about the intracoronary use of ACh, up to the point that its use is limited to experienced centers, and it is still off-label. In some countries, ACh does not have a clear indication for coronary administration due to safety concerns.

Our systematic review summarizes the reported side effects in the literature and clearly shows a low incidence of adverse events during provocative tests, accounting for a frequency of 0.5% event per test; death and permanent damages have notably never been reported.

Another very relevant aspect of this systematic review is that the incidence of adverse effects related to the use of ACh in MINOCA patients (patients, by definition, more hemodynamically unstable) do not present a higher rate of events than INOCA patients. In fact, the incidence of adverse events in these patients is statistically lower than in stable patients.

## 5. Conclusions

**Figure 3:**
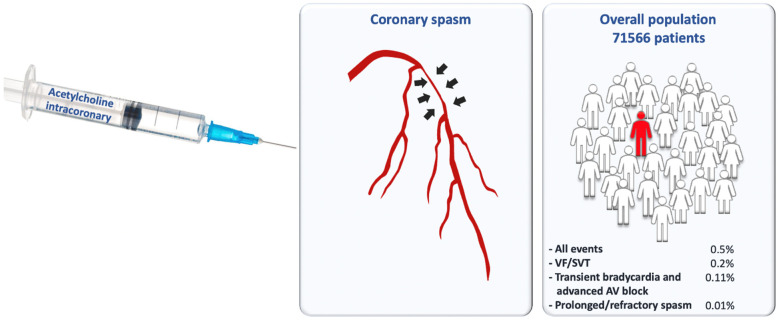
Central illustration: incidence of adverse events in patients evaluated with coronary acetylcholine.

To the best of our knowledge, this is the largest cohort of patients in which the safety profile of intracoronary ACh administration has been investigated. In addition, our results are in accordance with the aforementioned data, showing that the intracoronary administration of ACh in the setting of coronary spasm provocation testing can be performed safely despite its association with low rates of non-fatal side effects, as it plays a central diagnostic and prognostic role in coronary vasomotion disorders (Figure 3).

## Figures and Tables

**Table 1:** Randomized controlled trials with intracoronary infusion of acetylcholine.

First Author	Year of Publication	Type of Study	Study Population	Diagnosis	ACh Route of Administration	ACh Dosage	Side Effects, *n* of Patients	Kinds of Side Effect, Type (*n*)
Ford, T.J. [14]	2018	RCT	151	INOCA	Intracoronary	Incremental doses of 10^−6^, 10^−5^, and 10^−4^ mol/L	9	Persistent AF (1); paroxysmal AF (8).
Gomez-Lara, J. [15]	2018	RCT	63	CCS	Intracoronary	Incremental doses of 10^−6^ M and 10^−4^ M	0	0
Corcoran, D. [16]	2018	RCT	75	CCS	Intracoronary	10^−6^, 10^−5^, and 10^−4^ mol/L	0	0
Aoki, Y. [17]	2016	RCT	40	CCS	Intracoronary	Incremental doses of ACh at 10^−8^ and 10^−7^ mol/L f	0	0
Mischie, A.N. [18]	2013	RCT	24	CCS	Intracoronary	10^−5^ mol/L	2	Temporary asystole (less than 5 s) that recovered spontaneously (2).
Belkacemi, A. [19]	2012	RCT	150	CCS	Intracoronary	Incremental doses of 10^−6^, 10^−5^, and 10^−4^ mol/L/mL	0	0
Kim, J.W. [20]	2009	RCT	55	CCS	Intracoronary	Incremental doses of 10 μg, 20 μg, 50 μg, and 100 μg	0	0
Lüscher, T.F. [21]	2009	RCT	454	CCS	Intracoronary	10^−6^ to 10^−4^	2	Diffuse coronary vasoconstriction with marked hemodynamic consequences requiring resuscitation (1); MI (1).
Yasue, H. [22]	2008	RCT	78	INOCA	Intracoronary	Incremental doses of 50 and 100 μg in LCA, 50 μg in RCA	0	0
ENCORE investigators [23]	2003	RCT	343	CCS	Intracoronary	2 mL/min for 3 min acetylcholine 0.36, 3.6, and 18 mcg/mL	2	Diffuse vasoconstriction with hemodynamic consequences requiring resuscitation, in both cases without sequelae (2).
Azevedo, E.R. [24]	2001	RCT	20	CCS	Intracoronary	10^−4^ mol/L	2	Bradycardia/2nd degree AV block requiring no temporary pacing or atropine.
Hambrecht, R. [25]	2000	RCT	19	CCS	Intracoronary	Increasing doses of 0.072, 0.72, and 7.2 mcg	0	0
Lerman, A. [26]	1998	RCT	26	Non obstructive CAD	Intracoronary	10^−6^ to 10^−4^ mol/L)	0	0
Treasure, C.B. [27]	1995	RCT	23	CCS	Intracoronary	Incremental doses of 10^−9^ M, 10^−8^ M, 10^−7^ M, and 10^−6^ M	0	0

Abbreviations: RCT: randomized controlled trial; INOCA: ischemia with no-obstructive coronary arteries; AF: atrial fibrillation; CT: clinical trial; CCS: chronic coronary syndrome; LCA: left coronary artery; RCA: right coronary artery; AV: atrio-ventricular; MI: myocardial infarction; CAD: coronary artery disease.

**Table 2:** Adverse events reported in the included studies.

Adverse Event	RCT, *n* = 1521 Patients *n*, (%)	Observational Studies, *n* = 70,045 Patients *n*, (%)	Overall Population, *n* = 71,566 Patients *n*, (%)
All events	17, (1.12)	365, (0.5)	382, (0.5)
VF/SVT	0, (0)	148, (0.21)	148, (0.20)
AF	9, (0.59)	102, (0.14)	111, (0.15)
Transient bradycardia and advanced AV block	4, (0.26)	78, (0.11)	82, (0.11)
Prolonged/refractory spasm	3, (0.19)	9, (0.01)	12, (0.01)
NSVT	0, (0)	10, (0.01)	10, (0.01)
Hypotension	0, (0)	8, (0.01)	8, (0.01)
Shock	0, (0)	6, (<0.01)	6, (<0.01)
MI	1, (0.06)	4, (<0.01)	5, (<0.01)
Death	0, (0)	0, (0)	0, (0)

Abbreviations: VF/SVT: ventricular fibrillation, supraventricular tachycardia; AF: atrial fibrillation; AV: atrio-ventricular; NSVT: non-sustained ventricular tachycardia; MI: myocardial infarction.

**Table 3:** Recommendations for provocative coronary spasm testing.

Society	Recommendations	Class and Level of Evidence
2015 COVADIS [1].	Suspected history of VSA without documented episode, especially if nitrate-responsive rest angina, and/or marked diurnal variation in symptom onset/exercise tolerance, and/or rest angina without obstructive coronary artery disease. Unresponsive to empiric therapy. Acute coronary syndrome presentation in the absence of a culprit lesion. Unexplained resuscitated cardiac arrest. Unexplained syncope with antecedent chest pain. Recurrent rest angina following angiographically successful PCI.	I
	Invasive testing for non-invasive diagnosed patients unresponsive to drug therapy. Documented spontaneous episode of VSA to determine the ‘site and mode’ of spasm.	IIa
	Invasive testing for non-invasive diagnosed patients responsive to drug therapy.	IIb
	Emergent acute coronary syndrome. Severe fixed multi-vessel coronary artery disease including left main stenosis. Severe myocardial dysfunction (Class IIb if symptoms suggestive of vasospasm). Patients without any symptoms suggestive of VSA.	III
2013 JCS [8].	ACh provocation test during coronary angiography performed in patients in whom vasospastic angina is suspected based on symptoms, but who have not been diagnosed with coronary spasm by non-invasive evaluation.	I
	ACh provocation test during coronary angiography performed in patients who have been diagnosed with coronary spasm by non-invasive evaluation, and in whom medical treatment is ineffective or insufficiently effective.	IIa
	ACh provocation test during coronary angiography performed in patients who have been diagnosed with coronary spasm by non-invasive evaluation, and in whom medical treatment has been proven to be effective.	IIb
	ACh provocation test during coronary angiography performed in patients without symptoms suggestive of vasospastic angina. ACh provocation test during coronary angiography performed in patients who are considered at high risk of suffering a life-threatening complication of induced coronary spasm (e.g., patients with left main coronary trunk lesions; those with multivessel coronary lesions, including obstructive lesions; those with severe cardiac dysfunction; those with untreated congestive heart failure). However, in cases in which the onset of severe cardiac dysfunction or congestive heart failure may be a consequence of coronary spasm, the criteria for Class IIb apply). ACh provocation test during emergent coronary angiography performed in patients with acute coronary syndrome.	III
2013 ESC Stable coronary CAD [38]	Intracoronary provocative testing should be considered to identify coronary spasm in patients with normal findings or non-obstructive lesions on coronary arteriography and the clinical picture of coronary spasm to diagnose the site and mode of spasm.	IIa, C
2014 AHA/ACC, NSTE-ACS [39]	Provocative testing during invasive coronary angiography may be considered in patients with suspected vasospastic angina when clinical criteria and non-invasive testing fail to establish the diagnosis.	IIb, B

## Data Availability

Data available in a publicly accessible repository. The data presented in this study are openly available in PubMed.

## Supplementary Materials

The following are available online at https://www.mdpi.com/article/10.3390/jcm11041129/s1, Table S1: quality assessment with MINORS criteria.
