# Supplementary material for: Acetylcholine Use in Modern Cardiac Catheterization Laboratories: A Systematic Review
Source: J Clin Med. 2022 Feb 21;11(4):1129. doi: 10.3390/jcm11041129 (PMC8880288; doi:10.3390/jcm11041129)
Supplement: Supplementary file 1 [file jcm-11-01129-s001.zip › jcm-1580092-supplementary.pdf]

Supplementary Definitions

- Atrial fibrillation (AF) during intracoronary ACh provocation test was defined as documented AF on ECG monitoring during the provocation test in patients without AF before the procedure. Paroxysmal AF can be sub-classified in lasting up to the end of the procedure, < 48h, > 48h
- Bradycardia was defined as sinus rhythm with a rate below 50 beats per minute not necessarily requiring drugs or artificial pacing, but often going away with the interruption of ACh infusion.
- Myocardial infarction with non-obstructive coronary arteries (MINOCA) can be defined as a clinical syndrome characterized by evidence of myocardial infarction (myocardial injury with positive cardiac biomarker and clinical/EKG evidence of infarction) with no significant epicardial coronary arteries stenosis on angiography, in the absence of clear non-coronary causes of MI.
- Ischemia with non-obstructive coronary arteries (INOCA) can be defined as a clinical syndrome characterized by signs and symptoms of ischemic heart disease but found to have non-obstructed coronary epicardial arteries.

Table S1: quality assessment with MINORS criteria

Q: question. In evrery item the following points were assigned: “Not reported (0 point)", "Reported but inadequate (1 point), or "Reported and adequate (2 point)". Q1:A clearly stated aim; Q2: Inclusion of consecutive patient; Q3: Prospective collection of data; Q4: Endpoints appropriate to the aim of the study; Q5: Unbiased assessment of the study endpoint; Q6: Follow-up period appropriate to the aim of the study; Q7: Loss to follow up less than 5%; Q8: Prospective calculation of the study size; Q9: Adequate statistical analyses.

**References**	**Q1**	**Q2**	**Q3**	**Q4**	**Q5**	**Q6**	**Q7**	**Q8**	**Q9**	**Total**
Mohri M	2	2	2	2	2	2	0	0	2	14
Suda A	2	2	2	2	2	2	0	0	2	14
Isogai T	2	0	1	2	2	2	0	0	2	11
Choi BG	2	0	2	2	2	2	0	1	2	13
Sueda S	2	1	1	2	2	2	0	1	2	13
Ishii M	2	2	1	2	2	2	0	0	2	13
Park JY	2	2	1	2	2	2	0	0	2	13
Sara JD	2	0	1	2	2	2	0	0	2	11
Choi BG	2	2	2	2	2	2	0	2	2	16
Aziz A	2	2	0	2	2	2	0	0	2	12
Suzuki H	2	0	1	2	2	2	0	1	2	12
Akasaka T	2	2	1	2	2	2	0	1	2	14
Ong P	2	2	1	2	2	2	0	1	2	14
Takagi Y	2	1	0	2	2	2	0	0	2	11
Li YJ	2	1	1	2	2	2	0	1	2	13
Morita S	2	2	1	2	2	2	0	1	2	14
Lavi S	2	2	1	2	2	2	0	1	2	14
Sato K	2	2	0	2	2	2	0	0	2	12
Zaya M	2	1	1	2	2	2	0	1	2	13
Schoenenberger AW	2	2	2	2	2	2	0	2	2	16
Kaikita K	2	2	1	2	2	2	0	1	2	14
Choi CU	2	1	1	2	2	2	0	1	2	13
Kohno T	2	2	2	2	2	2	0	2	2	16
Kitano D	2	1	1	2	2	2	0	1	2	13
Kim MN	2	1	2	2	2	2	0	2	2	15
Arrebola-Moreno AL	2	1	1	2	2	2	0	1	2	13
Tateishi K	2	0	0	2	2	2	0	0	2	10
Im SI	2	2	2	2	2	2	0	1	2	15
Nakayama M	2	2	0	2	2	2	0	0	2	12
Nishino M	2	2	0	2	2	2	0	0	2	12
Suzuki S	2	2	0	2	2	2	0	0	2	12
Ohba K	2	2	2	2	2	2	2	2	2	18
Takae M	2	2	0	2	2	2	0	0	2	12
Fujimoto H	2	1	0	2	2	2	0	0	2	11

Ohura-Kajitani S	2	0	0	2	2	2	0	0	2	10
Tio RA	2	1	0	2	2	2	0	0	2	11
Halcox JP	2	0	0	2	2	2	0	0	2	10
Kurabayashi M	2	2	0	2	2	2	0	0	2	12
**References**	**Q1**	**Q2**	**Q3**	**Q4**	**Q5**	**Q6**	**Q7**	**Q8**	**Q9**	**Total**
Hoshino M	2	2	0	2	2	2	0	0	2	12
Yilmaz A	2	0	0	2	2	2	0	0	2	10
Maas R	2	0	0	2	2	2	0	0	2	10
Masumoto A	2	2	1	2	2	2	0	0	2	13
Pargaonkar VS	2	0	2	2	2	2	0	2	2	14
Asselbergs FW	2	0	0	2	2	2	0	0	2	10
Kim JW	2	0	0	2	2	2	0	0	2	10
AlBadri A	2	1	2	2	2	2	0	1	2	14
Sueda S	2	0	0	2	2	2	0	0	2	10
Kosugi M	2	2	0	2	2	2	0	2	2	14
Wakabayashi K	2	2	0	2	2	2	0	1	2	13
Teragawa H	2	0	0	2	2	2	0	0	2	10
AlBadri A	2	1	2	2	2	2	0	2	2	15
Nakayama N	2	2	1	2	2	2	0	1	2	14
Xiang DC	2	0	0	2	2	2	0	0	2	10
Nakao K	2	2	0	2	2	2	0	0	2	12
Takefuji M	2	1	0	2	2	2	0	0	2	11
Hasdai D	2	1	2	2	2	2	0	2	2	15
Ogawa T	2	1	1	2	2	2	0	1	2	13
Mizuno Y	2	0	0	2	2	2	0	0	2	10
Kurabayashi M	2	1	0	2	2	2	0	1	2	12
Itoh T	2	2	0	2	2	2	9	0	2	21
Odaka Y	2	2	0	2	2	2	0	2	2	14
Parikh RV	2	0	0	2	2	2	0	0	2	10
Hwang HJ	2	2	0	2	2	2	0	0	2	12
Yamanaga K	2	2	0	2	2	2	0	0	2	12
Di Fiore DP	2	1	1	2	2	2	0	1	2	13
Yang EH	2	0	0	2	2	2	0	0	2	10
Kaku B	2	1	0	2	2	2	0	1	2	12
Kaneda H	2	0	0	2	2	2	0	0	2	10
Okumura K	2	0	0	2	2	2	0	0	2	10
Pargaonkar VS	2	1	0	2	2	2	0	0	2	11
AlBadri A	2	1	0	2	2	2	0	0	2	11
Schächinger V	2	2	2	2	2	2	0	2	2	16
Sueda S	2	1	0	2	2	2	0	0	2	11
Lee BK	2	1	0	2	2	2	0	0	2	11
Konishi M	2	1	0	2	2	2	0	0	2	11
Funayama A	2	1	0	2	2	2	0	0	2	11
Sun H	2	2	0	2	2	2	0	0	2	12

Satoh S	2	1	0	2	2	2	0	0	2	11
Kim JW	2	1	2	2	2	2	0	2	2	15
Ong P	2	2	2	2	2	2	1	2	2	17
Kim JW	2	1	2	2	2	2	1	2	2	16
**References**	**Q1**	**Q2**	**Q3**	**Q4**	**Q5**	**Q6**	**Q7**	**Q8**	**Q9**	**Total**
Won H	2	1	1	2	2	2	0	1	2	13
Inamura Y	2	2	0	2	2	2	0	0	2	12
Sato A	2	2	0	2	2	2	0	0	2	12
Teragawa H	2	1	0	2	2	2	0	0	2	11
Tani S	2	0	0	2	2	2	0	0	2	10
Anderson TJ	2	2	2	2	2	2	0	0	2	14
Watanabe K	2	2	0	2	2	2	0	0	2	12
Vita JA	2	0	0	2	2	2	0	0	2	10
Sakata K	2	0	2	2	2	2	2	0	2	14
Hokimoto S	2	2	0	1	2	2	2	0	2	13
Suzuki M	2	2	0	2	2	2	0	0	2	12
Tsuburaya R	2	0	2	2	2	2	0	0	2	12
Houghton JL	2	0	2	2	2	2	0	0	2	12
Pirozzolo G	2	2	0	2	2	2	0	0	2	12
Matsumoto T	2	0	0	2	2	2	0	0	2	10
Park SJ	2	0	0	2	2	2	0	0	2	10
Xiang DC	2	0	0	2	2	2	0	0	2	10
Sueda S	2	0	0	2	2	2	0	0	2	10
Herrmann J	2	0	0	2	2	2	0	0	2	10
González-Costello J	2	0	0	2	2	2	0	0	2	10
Yilmaz A	2	0	2	2	2	2	0	0	2	12
Oumi T	2	2	2	1	2	2	0	0	2	13
Chu G	2	2	0	2	2	2	2	0	2	14
Kugiyama K	2	2	0	2	2	2	0	0	2	12
Rueda-Clausen CF	2	0	0	2	2	2	0	0	2	10
Montone RA	2	2	2	2	2	2	2	0	2	16
Corban MT	2	0	2	1	2	2	0	0	2	11
Sueda S	2	2	0	2	2	2	0	0	2	12
Hokimoto S	2	2	0	1	2	2	0	0	2	11
Haruta S	2	2	0	1	2	2	0	0	2	11
Chen KY	2	2	1	2	2	2	0	0	2	13
Takase B	2	2	0	1	2	2	0	0	2	11
Forte L	2	0	0	2	2	2	0	0	2	10
Yasue H	2	0	0	2	2	2	0	0	2	10
Morita S	2	2	2	2	2	2	2	2	2	18
Sueda S	2	2	0	2	2	2	2	0	2	14
Cannon RO	2	0	0	2	2	2	0	0	2	10
Honda T	2	0	0	2	2	2	0	0	2	10
Yamane T	2	2	0	2	2	2	0	0	2	12

Anderson TJ	2	0	0	2	2	2	0	0	2	10
Mitsutake Y	2	0	0	2	2	2	0	0	2	10
Nishio S	2	2	2	2	2	2	0	0	2	14
Hwang HJ	2	0	0	2	2	2	0	0	2	10
**References**	**Q1**	**Q2**	**Q3**	**Q4**	**Q5**	**Q6**	**Q7**	**Q8**	**Q9**	**Total**
Aizawa Y	2	0	0	2	2	2	0	0	2	10
Miwa K	2	2	0	2	2	2	0	0	2	12
Tani S	2	0	0	2	2	2	0	0	2	10
Miwa K	1	2	0	1	2	2	0	0	2	10
Fumimoto T	2	2	0	2	2	2	0	0	2	12
Uemura T	1	2	0	1	2	2	0	0	2	10
Nishizaki M	1	2	1	1	2	2	0	0	2	11
Suzuki M	2	2	2	2	2	2	0	0	2	14
Matsumoto T	2	0	0	2	2	2	0	0	2	10
Nanbu T	2	0	0	2	2	2	0	0	2	10
Miwa K	2	2	0	1	2	2	0	0	2	11
Katoh D	2	2	0	2	2	2	0	0	2	12
Mizuno Y	2	0	0	2	2	2	0	0	2	10
Sun H	2	2	2	2	2	2	0	0	2	14
Nishiyama K	2	0	0	2	2	2	0	0	2	10
Sawano M	2	2	2	2	2	2	2	2	2	18
Chauhan A	2	0	0	2	2	2	0	0	2	10
Kim JH	1	0	2	1	2	2	0	0	2	10
Oshima S	2	0	0	2	2	2	0	0	2	10
Lee SJ	2	0	0	2	2	2	0	0	2	10
Quyyumi AA	2	0	0	2	2	2	0	0	2	10
Kawakami T	2	2	0	1	2	2	0	0	2	11
Kaku B	2	0	0	2	2	2	0	0	2	10
Yoneyama K	2	0	0	2	2	2	0	0	2	10
Morita H	2	2	0	1	2	2	0	0	2	11
Takagi A	1	2	0	1	2	2	0	0	2	10
Anderson TJ	2	2	2	1	2	2	0	0	2	13
Yoneyama K	2	0	0	2	2	2	0	0	2	10
Kodama-Takahashi K	2	2	0	2	2	2	0	0	2	12
Sheikh AR	2	0	0	1	2	2	2	0	2	11
Fujii H	2	0	0	2	2	2	0	0	2	10
Nakagawa H	2	0	0	2	2	2	0	0	2	10
Naber ChK	1	2	0	1	2	2	0	0	2	10
Verna E	2	0	0	2	2	2	0	0	2	10
Komatsu M	2	2	2	1	2	2	0	0	2	13
Teragawa H	2	0	0	2	2	2	0	0	2	10
Mende A	2	2	0	2	2	2	0	0	2	12
Tanabe Y	2	2	0	2	2	2	0	0	2	12
Saito S	2	0	0	2	2	2	0	0	2	10

Shirai K	2	0	0	2	2	2	0	0	2	10
Miyamoto S	2	0	0	2	2	2	0	0	2	10
Ong P	2	2	0	2	2	2	0	0	2	12
Kumar A	2	0	2	1	2	2	0	0	2	11
**References**	**Q1**	**Q2**	**Q3**	**Q4**	**Q5**	**Q6**	**Q7**	**Q8**	**Q9**	**Total**
Okumura K	2	0	0	2	2	2	0	0	2	10
Elbaz M	2	0	0	2	2	2	0	0	2	10
Kirigaya H	2	0	0	2	2	2	0	0	2	10
Miwa K	2	0	0	2	2	2	0	0	2	10
Sakata K	2	0	2	2	2	2	2	0	2	14
Tamura A	2	2	2	2	2	2	2	0	2	16
Ninomiya Y	2	0	0	2	2	2	0	0	2	10
Bugiardini R	2	0	2	2	2	2	2	0	2	14
Cannon RO	2	2	0	2	2	2	0	0	2	12
Thanyasiri P	2	0	2	2	2	2	0	0	2	12
Inami T	2	2	2	2	2	2	2	2	2	18
Kodama K	2	1	0	2	2	2	0	0	2	11
Rahman H	2	2	1	2	2	2	0	0	2	13
Tani S	2	2	0	2	2	2	2	0	2	14
Miyamoto S	2	2	0	2	2	2	0	0	2	12
Sueda S	2	2	0	2	2	2	0	0	2	12
Qin Q	2	0	0	2	2	2	0	0	2	10
Sutani Y	2	0	0	2	2	2	2	0	2	12
Hitsumoto T	2	0	0	2	2	2	0	0	2	10
Sabaté M	2	0	2	2	2	2	2	0	2	14
Halcox JP	2	0	0	2	2	2	0	0	2	10
Houghton JL	2	0	2	2	2	2	0	0	2	12
Zeiher AM	2	0	0	2	2	2	0	0	2	10
Sumida H	2	2	0	2	2	2	0	0	2	12
Higuma T	2	0	2	2	2	2	1	0	2	13
Seo SM	2	0	0	2	2	2	2	0	2	12
Kugiyama K	2	2	0	2	2	2	0	0	2	12
Morikawa Y	2	2	0	2	2	2	0	0	2	12
Takano H	2	0	0	2	2	2	0	0	2	10
Okumura K	2	0	0	2	2	2	0	0	2	10
Yilmaz A	2	2	2	2	2	2	0	0	2	14
Matsuda K	2	0	0	2	2	2	0	0	2	10
Fang JC	2	0	0	2	2	2	0	0	2	10
Kodama K	2	0	0	2	2	2	0	0	2	10
Verna E	2	0	0	2	2	2	0	0	2	10
Raichlin E	2	2	0	2	2	2	0	0	2	12
Watanabe N	2	0	0	2	2	2	0	0	2	10
Yamakawa R	2	0	0	1	2	2	2	0	2	11
Chibana H	2	0	1	2	2	2	2	0	2	13

Pristipino C	2	0	0	2	2	2	0	0	2	10
Sakata K	2	2	0	2	2	2	2	0	2	14
Miwa K	2	2	0	2	2	2	0	0	2	12
Okumura K	2	0	0	2	2	2	0	0	2	10
**References**	**Q1**	**Q2**	**Q3**	**Q4**	**Q5**	**Q6**	**Q7**	**Q8**	**Q9**	**Total**
Nakajima K	2	0	0	2	2	2	2	0	2	12
Horio Y	2	0	0	2	2	2	0	0	2	10
Okumura K	2	2	2	2	2	2	0	0	2	14
Kugiyama K	2	2	0	2	2	2	0	0	2	12
Ioka T	2	0	0	2	2	2	0	0	2	10
Penny WF	2	0	0	2	2	2	0	0	2	10
Tanabe K	2	2	0	2	2	2	0	0	2	12
Sueda S	2	1	1	2	2	2	0	0	2	12
Miyawaki R	2	0	0	2	2	2	0	0	2	10
Monnink SH	2	0	0	2	2	2	0	0	2	10
Otsuji S	2	0	0	2	2	2	0	0	2	10
Nitenberg A	2	2	0	2	2	2	0	0	2	12
Roura G	2	0	0	2	2	2	0	0	2	10
Brugaletta S	2	0	0	2	2	2	2	0	2	12
Kurisu S	2	0	0	2	2	2	2	0	2	12
Summers MR	2	2	2	2	2	2	2	0	2	16
Sarno G	2	0	2	2	2	2	2	0	2	14
Yoshida K	2	0	0	2	2	2	0	0	2	10
Kugiyama K	2	2	0	2	2	2	0	0	2	12
Kugiyama K	2	0	0	2	2	2	0	0	2	10
Nishimura RA	2	0	0	2	2	2	0	0	2	10
Kato Y	2	0	0	2	2	2	0	0	2	10
Di Mario C	2	0	0	2	2	2	0	0	2	10
Yamakawa R	2	0	0	2	2	2	0	0	2	10
Iràculis E	2	2	0	2	2	2	2	0	2	14
Broxterman RM	2	0	0	2	2	2	2	0	2	12
el-Tamimi H	2	2	0	2	2	2	0	0	2	12
Fukuda Y	2	0	0	2	2	2	0	0	2	10
Yasue H	2	0	0	2	2	2	0	0	2	10
Kugiyama K	2	0	0	2	2	2	0	0	2	10
Prasad A	2	0	0	2	2	2	0	0	2	10
Simaitis A	2	0	0	2	2	2	0	0	2	10
Teragawa H	2	0	0	2	2	2	0	0	2	10
Kuhn FE	2	0	0	2	2	2	0	0	2	10
Drexler H, Zeiher AM	2	0	0	2	2	2	0	0	2	10
Shin DI	2	0	2	2	2	2	2	0	2	14
Suzuki Y	2	0	0	2	2	2	0	0	2	10
Kadohira T	2	0	0	2	2	2	0	0	2	10
Zeiher AM	2	0	2	2	2	2	0	0	2	12

Matsuyama K	2	0	0	2	2	2	0	0	2	10
Dimitrow PP	2	0	2	2	2	2	2	0	2	14
Reddy KG	2	0	0	2	2	2	0	0	2	10
Pleiner J	2	0	0	2	2	2	0	0	2	10
**References**	**Q1**	**Q2**	**Q3**	**Q4**	**Q5**	**Q6**	**Q7**	**Q8**	**Q9**	**Total**
Kawashima T	2	2	0	2	2	2	0	0	2	12
Werns SW	2	0	0	2	2	2	0	0	2	10
Sakakibara M	2	2	0	2	2	2	0	0	2	12
Aizawa K	2	0	0	2	2	2	1	0	2	11
van den Heuvel AF	2	0	0	2	2	2	0	0	2	10
Matsue Y	2	0	2	2	2	2	2	0	2	14
Motz W	2	0	0	2	2	2	0	0	2	10
Fuke S	2	0	0	2	2	2	2	0	2	12
Deyama J	2	0	2	2	2	2	2	0	2	14
Yamamoto H	2	0	2	2	2	2	2	0	2	14
Ashikaga T	2	2	2	2	2	2	2	0	2	16
Zeiher AM	1	0	2	2	2	2	2	0	2	13
Hirano Y	2	0	2	2	2	2	2	0	2	14
Gordon JB	2	0	2	2	2	2	2	0	2	14
Deng YB	2	2	2	2	2	2	2	0	2	16
Kanazawa K	2	0	2	2	2	2	2	0	2	14
Masumoto A	2	2	2	2	2	2	2	0	2	16
Watanabe T	2	2	2	2	2	2	2	0	2	16
Fujii K	2	0	2	2	2	2	2	0	2	14
Kawano H	2	0	2	2	2	2	2	0	2	14
Lagerqvist B	2	0	2	2	2	2	2	0	2	14
Vaisrub S.	2	0	2	2	2	2	2	0	2	14
Eichstädt HW	2	0	2	2	2	2	2	0	2	14
Polvani G	2	0	2	2	2	2	2	0	2	14
Heras M	2	0	2	2	2	2	2	0	2	14
Webb CM	2	0	2	2	2	2	2	0	2	14
Egashira K	2	0	2	2	2	2	2	0	2	14
Kato M	2	0	2	2	2	2	2	0	2	14
Hu J	2	0	2	2	2	2	2	0	2	14
Raichlin E	2	0	2	2	2	2	2	0	2	14
Brugaletta S	2	2	2	2	2	2	2	0	2	16
Ludmer PL	2	0	2	2	2	2	2	0	2	14
Erne P	2	0	2	2	2	2	2	0	2	14
Tousoulis D	2	0	2	2	2	2	2	0	2	14
Egashira K	2	0	2	2	2	2	2	0	2	14
Mohri M	2	2	2	2	2	2	2	0	2	16
Drexler H	2	0	2	2	2	2	2	0	2	14
Miwa K	2	2	2	2	2	2	2	0	2	16
Shimizu H	2	0	2	2	2	2	2	0	2	14

Tousoulis D	2	2	2	2	2	2	2	0	2	16
Werner GS.	2	0	2	2	2	2	2	0	2	14
Klein RM.	2	0	2	2	2	2	2	0	2	14
Vassanelli C	2	0	2	2	2	2	2	0	2	14
**References**	**Q1**	**Q2**	**Q3**	**Q4**	**Q5**	**Q6**	**Q7**	**Q8**	**Q9**	**Total**
Baldus S	2	2	2	2	2	2	2	0	2	16
Toyo-oka T	2	0	2	2	2	2	2	0	2	14
Newman CM	2	0	2	2	2	2	2	0	2	14
Mikuniya A	2	0	2	2	2	2	2	0	2	14
Fujita H	2	0	2	2	2	2	2	0	2	14
Kaikita K	2	0	2	2	2	2	2	0	2	14
Vrints CJ	2	2	0	2	2	2	2	0	2	14
Aptecar E	2	0	2	2	2	2	2	0	2	14
Hillier C	2	0	2	2	2	2	2	0	2	14
Ferrari M	2	2	2	2	2	2	2	0	2	16
Miyata S	2	2	2	2	2	2	2	0	2	16
Vita JA	2	0	2	2	2	2	2	0	2	14
Nishijima H	2	0	2	2	2	2	2	0	2	14
Matsu-ura Y	2	0	2	2	2	2	2	0	2	14
Takeuchi M	2	0	2	2	2	2	2	0	2	14
Hofma SH	2	0	2	2	2	2	2	0	2	14
Brush JE Jr	2	0	2	2	2	2	2	0	2	14
Ogawa H	2	0	2	2	2	2	2	0	2	14
Dubois-Rande JL	2	0	2	2	2	2	2	0	2	14
Murase S	2	0	2	2	2	2	2	0	2	14
Riemekasten G	1	0	0	2	2	2	2	0	2	11
Etsuda H	2	2	2	2	2	2	2	0	2	16
Dubois-Randé JL	2	0	2	2	2	2	2	0	2	14
Miwa K	2	0	2	2	2	2	2	0	2	14
Sueda S	2	0	2	2	2	2	2	0	2	14
Fujita H	2	0	2	2	2	2	2	0	2	14
el-Tamimi H	2	2	2	2	2	2	2	0	2	16
Wöhrle J	2	0	2	2	2	2	2	0	2	14
Takagi Y	2	2	2	2	2	2	2	0	2	16
Thaning P	2	0	2	2	2	2	2	0	2	14
Nellessen U	2	0	2	2	2	2	2	0	2	14
Manginas A	2	0	2	2	2	2	2	0	2	14
Miwa K	2	0	2	2	2	2	2	0	2	14
Kugiyama K	2	0	2	2	2	2	2	0	2	14
Werner GS	2	0	2	2	2	2	2	0	2	14
Nitenberg A	2	0	2	2	2	2	2	0	2	14
Yoshimura M	2	0	2	2	2	2	2	0	2	14
Javadzadegan A	2	0	2	2	2	2	2	0	2	14
Kitani Y	2	2	2	2	2	2	2	0	2	16

Egashira K	2	0	2	2	2	2	2	0	2	14
Bund SJ	2	0	2	2	2	2	2	0	2	14
Cox ID	2	0	2	2	2	2	2	0	2	14
Kamiya H	2	0	2	2	2	2	2	0	2	14
**References**	**Q1**	**Q2**	**Q3**	**Q4**	**Q5**	**Q6**	**Q7**	**Q8**	**Q9**	**Total**
Egashira K	2	2	2	2	2	2	2	0	2	16
Aoki J	2	0	2	2	2	2	2	0	2	14
Takase H	2	0	2	2	2	2	2	0	2	14
Egashira K	2	0	2	2	2	2	2	0	2	14
Choi YH	2	0	0	2	2	2	2	0	2	12
El-Tamimi H	2	2	2	2	2	2	2	0	2	16
Mitsutake Y	2	0	2	2	2	2	2	0	2	14
Uchida Y	2	0	2	2	2	2	2	0	2	14
Collins P	2	0	2	2	2	2	2	0	2	14
Dubois-Randé JL	2	0	2	2	2	2	2	0	2	14
Hirooka Y	2	0	2	2	2	2	2	0	2	14
Kern MJ	2	0	0	2	2	2	0	0	2	10
Yamanishi K	2	0	0	2	2	2	0	0	2	10
Sueda S	2	0	0	2	2	2	0	0	2	10
Murakami H	2	0	0	2	2	2	0	0	2	10
Kawata M	2	0	0	2	2	2	0	0	2	10
Chinushi M	2	0	0	2	2	2	0	0	2	10
Matsumura M	2	0	0	2	2	2	0	0	2	10
Suzuki N	2	0	0	2	2	2	0	0	2	10
Angelini P	2	0	0	2	2	2	0	0	2	10
Munakata K	2	0	0	2	2	2	0	0	2	10
Kodama K	2	0	0	2	2	2	0	0	2	10
Tachibana K	2	0	0	2	2	2	0	0	2	10
Masaki N	2	0	0	2	2	2	0	0	2	10
Kuhn FE	2	0	0	2	2	2	0	0	2	10
Fujiwara Y	2	0	0	2	2	2	0	0	2	10
Sasaki T	2	0	0	2	2	2	0	0	2	10
Yamaki M	2	0	0	2	2	2	0	0	2	10
Wada M	2	0	0	2	2	2	0	0	2	10
Nishizaki M	2	0	0	2	2	2	0	0	2	10
Chinushi Y	2	0	0	2	2	2	0	0	2	10
Suzuki K	2	0	0	2	2	2	0	0	2	10
Tani S	2	0	0	2	2	2	0	0	2	10
Maeda N	2	0	0	2	2	2	0	0	2	10
Nardi F	2	0	0	2	2	2	0	0	2	10
Oyama N	2	0	0	2	2	2	0	0	2	10
Amanullah MR	2	0	0	2	2	2	0	0	2	10
Shimizu M	2	0	0	2	2	2	0	0	2	10
Adachi Y	2	0	0	2	2	2	0	0	2	10

Saito Y	2	0	0	2	2	2	0	0	2	10
Nakahashi T	2	0	0	2	2	2	0	0	2	10
Ohtaki Y	2	0	0	2	2	2	0	0	2	10
Horimoto M	2	0	0	2	2	2	0	0	2	10
**References**	**Q1**	**Q2**	**Q3**	**Q4**	**Q5**	**Q6**	**Q7**	**Q8**	**Q9**	**Total**
Ando S	2	0	0	2	2	2	0	0	2	10
Maejima Y	2	0	0	2	2	2	0	0	2	10
Ito M	2	0	0	2	2	2	0	0	2	10
Ogawa T	2	0	0	2	2	2	0	0	2	10
Fukuda N	2	0	0	2	2	2	0	0	2	10
Morino Y	2	0	0	2	2	2	0	0	2	10
Komukai K	2	0	0	2	2	2	0	0	2	10
Fuertes J	2	0	0	2	2	2	0	0	2	10
Hara Y	2	0	0	2	2	2	0	0	2	10
Wijpkema JS	2	0	0	2	2	2	0	0	2	10
Sekiya M	2	0	0	2	2	2	0	0	2	10
Shimizu K	2	0	0	2	2	2	0	0	2	10
Maekawa K	2	0	0	2	2	2	0	0	2	10
O-uchi J	2	0	0	2	2	2	0	0	2	10
Horimoto M	2	0	0	2	2	2	0	0	2	10
Horimoto M	2	0	0	2	2	2	0	0	2	10
Tanabe Y	2	0	0	2	2	2	0	0	2	10
Itoh E	2	0	0	2	2	2	0	0	2	10
Eshtehardi P	2	0	0	2	2	2	0	0	2	10
Adachi N	2	0	0	2	2	2	0	0	2	10
Takaoka K	2	0	0	2	2	2	0	0	2	10
Hiasa K	2	0	0	2	2	2	0	0	2	10
Mancio J	2	0	0	2	2	2	0	0	2	10
Horimoto M	2	0	0	2	2	2	0	0	2	10
Murakami H	2	0	0	2	2	2	0	0	2	10
Kaku B	2	0	0	2	2	2	0	0	2	10
Endoh Y	2	0	0	2	2	2	0	0	2	10
Osaki J	2	0	0	2	2	2	0	0	2	10
Utsunomiya D	2	0	0	2	2	2	0	0	2	10
Tanaka Y	2	0	0	2	2	2	0	0	2	10
Horimoto M	2	0	0	2	2	2	0	0	2	10
Treasure CB	2	0	0	2	2	2	0	0	2	10
Sakata K	2	0	0	2	2	2	0	0	2	10
Suzuki S	2	0	0	2	2	2	0	0	2	10
Cannan CR	2	0	0	2	2	2	0	0	2	10
Nakagawa T	2	0	0	2	2	2	0	0	2	10
Ishikura M	2	0	0	2	2	2	0	0	2	10
Takeda M	2	0	0	2	2	2	0	0	2	10
Suzuki M	2	0	0	2	2	2	0	0	2	10

Ito S	2	0	0	2	2	2	0	0	2	10
Hishikari K	2	0	0	2	2	2	0	0	2	10
Burns A	2	0	0	2	2	2	0	0	2	10
Nii T	2	0	0	2	2	2	0	0	2	10
**References**	**Q1**	**Q2**	**Q3**	**Q4**	**Q5**	**Q6**	**Q7**	**Q8**	**Q9**	**Total**
Kobayashi N	2	0	0	2	2	2	0	0	2	10
Tsujita K	2	0	0	2	2	2	0	0	2	10
Yamasa T	2	0	0	2	2	2	0	0	2	10
Oshima Y	2	0	0	2	2	2	0	0	2	10
Hendriks ML	2	0	0	2	2	2	0	0	2	10
Maekawa K	2	0	0	2	2	2	0	0	2	10
Giordan M	2	0	0	2	2	2	0	0	2	10
Kiuchi K	2	0	0	2	2	2	0	0	2	10
Sueda S	2	0	0	2	2	2	0	0	2	10
